# Using the Translational Science Benefits Model to assess the impact of the Penn Implementation Science Center in Cancer Control

**DOI:** 10.1017/cts.2024.554

**Published:** 2024-10-16

**Authors:** Robert Schnoll, Justin E. Bekelman, Daniel Blumenthal, David A. Asch, Alison M. Buttenheim, Krisda H. Chaiyachati, Susan M. Domchek, Oluwadamilola M. Fayanju, Peter Gabriel, Brian P. Jenssen, Frank T. Leone, Anne Marie McCarthy, Katherine L. Nathanson, Ravi B. Parikh, Katharine A. Rendle, Rachel C. Shelton, Lawrence N. Shulman, Samuel U. Takvorian, Susan Ware, E. Paul Wileyto, Rinad S. Beidas

**Affiliations:** 1 Department of Psychiatry, Perelman School of Medicine, University of Pennsylvania, Philadelphia, USA; 2 Abramson Cancer Center, Perelman School of Medicine, University of Pennsylvania, Philadelphia, USA; 3 Penn Center for Cancer Care Innovation, University of Pennsylvania, Philadelphia, USA; 4 Department of Medicine, Perelman School of Medicine, University of Pennsylvania, Philadelphia, USA; 5 Department of Family and Community Health, School of Nursing, University of Pennsylvania, Philadelphia, USA; 6 Verily Life Sciences, South San Francisco, CA; 7 Basser Center for BRCA, Perelman School of Medicine, University of Pennsylvania, Philadelphia, USA; 8 Department of Surgery, Perelman School of Medicine, University of Pennsylvania, Philadelphia, USA; 9 Department of Pediatrics, Perelman School of Medicine, University of Pennsylvania, Philadelphia, USA; 10 Pulmonary, Allergy & Critical Care, Perelman School of Medicine, University of Pennsylvania, Philadelphia, USA; 11 Center for Clinical Epidemiology and Biostatistics, Perelman School of Medicine, University of Pennsylvania, Philadelphia, USA; 12 Department of Family Medicine and Community Health, Perelman School of Medicine, University of Pennsylvania, Philadelphia, USA; 13 Department of Sociomedical Sciences, Mailman School of Public Health, Columbia University, New York, USA; 14 Department of Medical Social Sciences, Feinberg School of Medicine, Northwestern University, Chicago, USA

**Keywords:** Translational Science Benefits Model, impact, implementation science, cancer, behavioral economics

## Abstract

Traditional approaches for evaluating the impact of scientific research – mainly scholarship (i.e., publications, presentations) and grant funding – fail to capture the full extent of contributions that come from larger scientific initiatives. The Translational Science Benefits Model (TSBM) was developed to support more comprehensive evaluations of scientific endeavors, especially research designed to translate scientific discoveries into innovations in clinical or public health practice and policy-level changes. Here, we present the domains of the TSBM, including how it was expanded by researchers within the Implementation Science Centers in Cancer Control (ISC3) program supported by the National Cancer Institute. Next, we describe five studies supported by the Penn ISC3, each focused on testing implementation strategies informed by behavioral economics to reduce key practice gaps in the context of cancer care and identify how each study yields broader impacts consistent with TSBM domains. These indicators include *Capacity Building, Methods Development* (within the Implementation Field) and *Rapid Cycle Approaches*, implementing *Software Technologies*, and improving *Health Care Delivery* and *Health Care Accessibility*. The examples highlighted here can help guide other similar scientific initiatives to conceive and measure broader scientific impact to fully articulate the translation and effects of their work at the population level.

## Introduction

The National Cancer Institute (NCI) is charged with improving the lives of all Americans and supports cancer research, training, and education. Typically, NCI-funded scientists measure their impact through traditional metrics including publications, scientific presentations, and grant support. Recently, the NCI has also prioritized translating funded research into enhanced clinical treatments, public health practice, and public policy to prevent cancer, enhance treatments, and improve survivorship outcomes. Consistent with this translational focus, traditional metrics must also capture societal benefits from research [1].

Investigators at Washington University in St Louis’s Institute for Clinical and Translational Sciences developed a framework to more comprehensively assess scientific impact: the Translational Science Benefits Model (TSBM) [2]. Based on a literature review, a modified Delphi technique, and expert feedback, this model considers societal impact beyond traditional metrics. This novel approach is particularly useful for assessing the impact of translational research, which promotes “bench-to-bedside” outcomes. The TSBM outlines potential impacts, discoveries, and accomplishments that can more fully capture the societal and translational impact of science. It defines benefits in four general domains: Clinical and Medical, Community and Public Health, Economic, and Policy.

The TSBM is starting to be applied in the national Clinical and Translational Science Awards network [3] and the NCI’s Implementation Science Centers in Cancer Control (ISC3) program[4,5]. Implementation science focuses on the development and testing of strategies to close the gap between the availability and use of evidence-based interventions and policies [6]. Specifically, ISC3 supports the development, testing, and refinement of implementation strategies to promote the widespread, routine, and equitable use of evidence-based prevention and cancer control interventions [4,5]. Additionally, this initiative was designed to expand and strengthen the field of implementation science by training new investigators, building research infrastructure, and addressing systemic health inequities pervading gaps in cancer outcomes [7–9]. Seven groups, spanning universities and community organizations across the United States (US), were funded through ISC3.

One center is at the University of Pennsylvania, in collaboration with Northwestern University [4]. The Penn ISC3’s overarching objective is to apply insights from behavioral economics to rapidly accelerate the pace at which evidence-based practices for cancer care are deployed and delivered equitably, thereby increasing their reach among, and impact on, individuals with cancer. Behavioral economics characterizes how people routinely deviate from traditionally expected “utility maximizing” behavior due to predictable habits and heuristics [10–12]. Behavioral economic theory also proposes methods for changing behavior by adjusting the decision-making environment or “choice architecture” to facilitate evidence-based choices [13,14]. Subtle changes to the choice architecture (termed “nudges”), such as setting desired options as defaults and requiring decision-makers to opt out to act differently, have facilitated behavior change across health care domains [15,16].

The Penn ISC3 has launched five projects, each using rigorous methods to test nudges to increase a specific evidence-based intervention for improving cancer care or control: (1) tobacco use treatment; (2) serious illness conversations; (3) magnetic resonance imaging for early breast cancer detection among women with dense breasts; (4) genetic testing for breast and ovarian cancer; and (5) patient-reported outcome assessments. All studies share four key elements: (1) rapid cycle innovation approaches [17] to refine intervention designs through formative pretrial evaluations; (2) mixed-methods analyses to assess mechanisms of study impact; (3) a focus on understanding and addressing health inequities and disparities in outcomes; and (4) integration into the electronic medical record (EMR) to make clinical deployment efficient. Projects are based in an Implementation Science Laboratory (iLab) [8] containing the 12 Penn Medicine Abramson Cancer Center Service Line sites and multiple Penn Medicine primary care and gynecologic practices depending on a study’s focus.

In this paper, we describe the Penn ISC3’s work to demonstrate how the TSBM can conceptualize impact beyond traditional metrics. The ISC3 network modified the TSBM to include a fifth domain, referred to as Implementation Field, that includes potential impacts like developing new methods specific to implementation science (Methods Development), strengthening partner and practitioner capabilities, increasing mentor and mentee skills, and adaptations, for example, to address issues relevant to racial and ethnic equity (Table 1). While we did not engage in a formal process of mapping the results from our studies onto TSBM domains, all authors collectively sought to conceptualize the results from our studies within this expanded set of TSBM domains to illustrate the Penn ISC3’s impact through a broader viewpoint (Table 2; Supplement 1). Consensus was reached through an iterative process in drafting this paper and completing Table 2.

**Table 1.** Domains and Indicators of the Implementation Science in Cancer Control Centers Translational Science Benefits Model

**Implementation Field**	
*Methods and Measures*	
Measures Development	Developed new measures of implementation determinants, processes, or outcomes
Methods Development	Developed new methods for implementation strategy selection and optimization; developed new toolkits; identified summaries and literature gaps; developed new engagement strategies of costing tools
Raid Cycle Approaches	Rapid needs assessment and testing
Adaptation	Developed or adapted an implementation process or strategy with an explicit focus on health equity; developed measures of adaptation; tools to track adaptations
*Capacity Building*	
Building Partner or Practitioner Research Capacity	Partner led or participated on grants, publications, presentations
Engagement	Partner included in research study selection; dissemination of results to partners; increased diversity of partnerships
Build Implementation Science Research Capacity	Support early-career researchers; promote diversity of research community; broaden partnership capacity; develop tools to help researchers
**Clinical and Medical Benefits**	
*Procedures and Guidelines*	
Diagnostic Procedures	Methods and techniques performed to diagnose disease, disorders, or disability
Investigative Procedures	Research methods used in pre-clinical, clinical, and other scientific studies
Guidelines	Formal recommendations or principles to assist with patient care for specific clinical circumstances
Therapeutic Procedures	Methods and techniques concerned with interventions, treatment or prevention of diseases
*Tools and Products*	
Biological Factors & Products	Biological substances used to indicate, diagnose, prevent or treat diseases or medical conditions
Biomedical Technology	Technology applications for the solution of medical problems
Drugs	Pharmaceutical products for human or veterinary use
Equipment & Supplies	Apparatus, instruments, and materials for diagnostic, surgical, therapeutic, and scientific procedures
Software Technologies	Computer programs or software installed on mobile or other electronic devices
**Community & Public Health Good**	
*Health Activities and Products*	
Community Health Services	Diagnostic, therapeutic and preventive health services provided for individuals in the community
Consumer Software	Digital and mobile technologies used by or for consumers to improve health care delivery and outcomes
Health Education Resources	Educational resources that relate to improvement of health on a personal or community basis
*Healthcare Characteristics*	
Health Care Accessibility	Equity and ability for all to gain entry to and to receive services from the health care system
Health Care Delivery	Provision and distribution of health services to a patient population
Health Care Quality	General characteristics of the health service or care provided based on accepted standards of quality
*Health Promotion*	
Disease Prevention & Reduction	Resources that enhance health promotion and disease prevention in communities or population
Life Expectancy & Quality of Life	Life expectancy or quality of life for communities or populations
Public Health Practices	Organization or delivery of public health services benefits to communities or population

**Economic Benefits**	
*Commercial Products*	
License Agreements	Governmental permit based on intellectual property
Nonprofit or Commercial Entities	Creation of businesses or nonprofit entities
Patents	Government authority or license based on intellectual property
*Financial Savings and Benefits*	
Cost Effectiveness	Improvement in the benefits of a program relative to its costs
Cost Savings	Reduced financial costs of services or goods to providers or consumers
Societal & Financial Cost of Illness	Reduced social and economic costs of acute or chronic disease or other health conditions
**Policy & Legislative Benefits**	
*Advisory Activities*	
Committee Participation	Participation in advisory, standards, or other governmental or nongovernmental committees
Expert Testimony	Presentation of data or results to governmental, judicial or other regulatory bodies
Scientific Research Reports	Research findings or summaries provided to inform policy or regulatory activity
*Policies and Legislation*	
Legislation	Bills, laws, statutes, and ordinances passed through governmental bodies
Policy	Procedural rules formally adopted by governmental bodies or other organizations
Standard	Formal industry, occupational or governmental procedures and rules

*Note*: Adapted from Luke et al. (2018) [2].

**Table 2.** Primary Translational Science Benefits Model indicators and examples addressed by the Penn Indicators of the Implementation Science in Cancer Control Centers projects

TSBM Domain and Indicator	Study 1: Tobacco Use Treatment	Study 2: Serious Illness Conversations (SICs)	Study 3: Supplemental Breast MRI	Study 4: Genetic Counseling	Study 5: Patient-Reported Outcomes (PROs)
Implementation Field: Methods Development or Rapid Cycle Approaches	Qualitative methods to develop nudges	Qualitative methods and usability testing to develop nudges	Qualitative methods and small pilot studies to develop nudges	Qualitative methods and small pilot studies to develop nudges	Qualitative methods to develop nudges and cancer center-wide surveys
Implementation Field: Capacity Building	Develop junior investigators and abilities of network partners	Develop junior investigators and abilities of network partners	Develop junior investigators and abilities of network partners	Support experienced investigators new to implementation science	Develop junior investigators; co-design strategies with triage nurses and informatics leaders
Community & Public Health Good: Healthcare Quality	Increased tobacco treatment	Increased number of documented SICs	Increased use of MRI for breast cancer early detection*	Increased use of genetic testing for breast and ovarian cancer risk*	Increased use of PROs*
Community & Public Health Good: Healthcare Accessibility	Clinician nudge equally effective across minoritized groups	Clinician and patient nudge equally effective across minoritized groups	Improved use of MRI across minoritized groups*	Improved use of genetic testing across minoritized groups*	Improved use of PROs across minoritized groups*
Clinical and Medical Benefits: Software Technologies	Develop two best practice alerts for tobacco use treatment and screening which have been scaled across the service line	Create text message priming survey for patients	Design workflows to deliver text messages to patients and embed clinician-directed nudges into mammogram reports	Build electronic phenotyping algorithm; implement “pend and send” clinician nudges	Redesign PRO monitoring workflows and launch new reports and dot-phrases in the electronic medical record

*Note:* denotes expected impacts following trial completion.

### Project 1: Promoting tobacco use treatment in oncology

**Figure 1.**
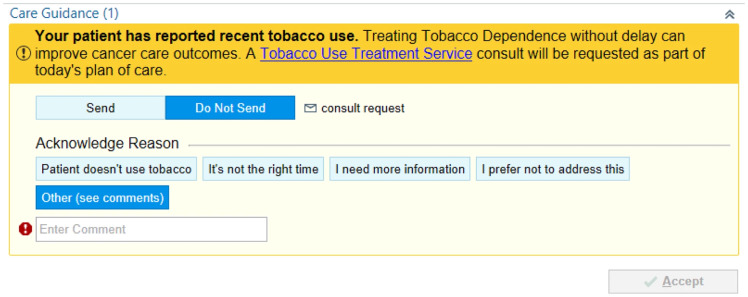
Tobacco use treatment clinician-directed nudge.

Continued tobacco use after a cancer diagnosis reduces the effectiveness of medical treatments and worsens clinical outcomes [18]. Yet, nearly half of cancer patients previously using tobacco continue doing so while receiving cancer care, and evidence-based tobacco cessation treatment is rarely provided [19]. Cognitive heuristics, commonly activated in stressful, complex situations, may impair engagement in evidence-based tobacco treatment [20–23]. In this cluster-randomized pragmatic clinical trial, we developed and evaluated messages (i.e., nudges), informed by behavioral economics, integrated into the EMR, and directed at patients and/or clinicians, to promote evidence-based tobacco use treatment (TUT). We examined patient sociodemographic information and clinic-level variables as potential moderators of nudge effectiveness. Patients were prompted via the health system portal to discuss tobacco use treatment with their clinician by emphasizing the importance of TUT to their care. Clinicians received a best practice alert emphasizing the value of TUT for their patients and featuring a default button to instantly refer patients to Tobacco Use Treatment Services (Figure 1). In the end, clinician-directed nudges tripled TUT engagement compared to usual care [24].

#### TSBM domains

This project’s development, implementation, and results illustrate several TSBM impacts. It demonstrates impact in *Methods Development* (within the Implementation Field vs. Investigative Procedures) by using formative research methods (key informant interviews and focus groups) to optimize patient- and clinician-directed messages. This work helped to identify the heuristics to be targeted, determine preferred wording, and inform message delivery mechanisms [25]. Under *Capacity Building*, this project supported iLab collaborators and augmented their ability to conduct pragmatic clinical trials and implement behavioral design methodologies. Additionally, the trial was co-led by an early-career investigator mentored by the grant’s multiple principal investigators (MPIs) and who subsequently received an R37 award to expand this work to address TUT in pediatrics. Third, under *Software Technologies*, two BPAs were activated in the EMR, and a patient-directed strategy was implemented through the patient portal, illustrating novel ways to deliver implementation strategies. Nudges facilitated over 500 patients being referred to TUT services or being prescribed tobacco use medication. Fourth, the results improved *Healthcare Quality*, in this case by increasing TUT engagement, an effect that was equitable across white and nonwhite study patients, supporting *Health Care Accessibility*. The team is collaborating with the health system to integrate clinician nudges into routine care, thereby *Improving Healthcare Delivery* via sustainable institutional *Policy Changes*.

### Project 2: Promoting the use of serious illness conversations (SICs) in oncology

Patients with cancer often receive medical care near the end of their lives that may not align with their values [26]. Serious illness conversations (SICs), which document patients’ care preferences, are an evidence-based method to reduce unwanted end-of-life medical treatments [27]. Unfortunately, most patients with cancer lack a documented SIC before their death [28]. SIC engagement is likely influenced by clinician heuristics (e.g., overestimating the life expectancy of patients with advanced cancer) and patient heuristics (e.g., normative beliefs concerning the appropriateness of SICs) [29,30]. In this cluster-randomized pragmatic trial, we evaluated the effect of patient and/or clinician messages designed to counter heuristics that undermine SICs and examined patient sociodemographic information and clinic-level variables as moderators of nudge effectiveness. Compared to the active control arm (consisting of identification of high-risk patients and opt-out text message reminders to complete SICs), participants in the combined patient and clinician message arm were significantly more likely to have documented SICs [31].

#### TSBM domains

**Figure 2.**
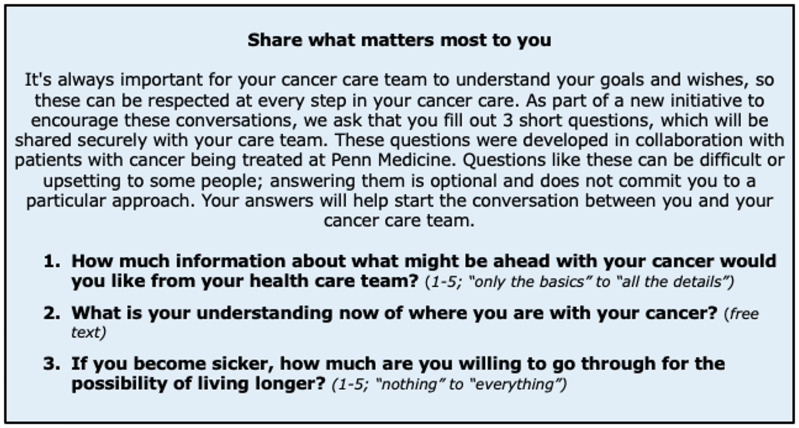
Patient-directed nudge to promote engagement with serious illness conversations.

Our study of implementation strategies to increase SICs further illustrates impact across TSBM domains. First, *Rapid Cycle Approaches* (RCAs) were used to optimize and de-risk implementation strategies prior to trial launch. The study team held meetings with behavioral design experts, discussions with oncology clinicians, and focus groups with patients and caregivers. Multiple rounds of usability testing with clinicians and patients helped finalize the content of the nudges. This process also resulted in an important *Adaptation* to our implementation process to address issues relevant to equity. We changed the patient message delivery channel from the patient portal to a text-messaging platform because fewer African American or Black individuals were registered users of our patient portal relative to our population of patients with cancer [32]. To address this gap, illustrating the *Software Technologies* domain, a new workflow was developed to send a more accessible survey to patients via text message that primed them to engage in SICs (Figure 2).

As with our tobacco project, our SIC study supported *Capacity Building* by involving iLab collaborators and helping them conduct implementation science pragmatic clinical trials and deploy innovation methods like rapid cycle approaches. Further, the trial was co-led by two early-career investigators who were mentored by MPIs. One of these early-career investigators received a K08 to expand the use of machine learning as a novel analytic method (*Methods Development*) to guide risk prediction algorithms for identifying cancer patients eligible for palliative care referrals. Lastly, the impact of the combined patient and clinician nudge arm on SIC rates improved *Healthcare Quality*, in this case by improving the frequency of documented serious illness conversations, and did so equitably across white and Black patients, supporting *Health Care Accessibility* for all.

### Project 3: Promoting the use of magnetic resonance imaging (MRI) for breast cancer early detection among women with dense breasts

About five million American women aged 40 to 74 years have extremely dense breasts, [33] a trait which poses higher breast cancer risk [34] and reduces mammogram sensitivity [35]. However, while accumulated analyses [36,37] suggest supplemental MRI can reduce interval cancers by up to 50% for these women, only about 10% of those eligible receive MRI [38]. Screening rates are even lower among Black and Hispanic women [39], representing a substantial practice gap and health inequity. Patient-level barriers to MRI screening include perceived financial burden, anxiety about undergoing MRI and receiving results, and lack of awareness of breast density [37,40–44]. Clinician barriers include the absence of consistent guidelines, worries about financial costs or potential false positives, and lack of awareness of the clinical importance of breast density or legislation mandating insurance coverage [33,37,45–47] These barriers yield uncertainty about breast MRI’s value, which can lead patients and clinicians to rely on heuristics [48] that reduce evidence-based care and exacerbate health inequities [49].

In October 2023, Penn ISC3 launched a trial [50] designed to increase MRI screening among women with extremely dense breasts using messages informed by behavioral economics to counter potential heuristics reducing breast MRI engagement. This 2x2 pragmatic, stepped wedge design compares text messages to patients and/or messages to clinicians (integrated into the mammogram report) vs. usual care for increasing the rate of ordering or scheduling supplemental breast MRI. Although this trial is ongoing, critical steps in the study design illustrate several TSBM domains.

#### TSBM domains

*RCAs* were used to finalize message content and design. Key informant interviews were held with experts in behavioral science, breast cancer screening, and health equity, guiding the targeted cognitive heuristic (omission bias), delivery channel (“impression” section of mammogram reports to integrate messages into the clinical workflow), and message content (wording to address heuristics and clinician education materials). For the patient message, the study team and a patient advisory committee helped refine content and procedures. Rapid pilot tests were then conducted with 180 patients to compare three messages reflecting different heuristics that may impede scheduling breast MRI. This formative work led to messages that addressed the availability heuristic and the development of web-based educational material, the latter representing a *Health Education Resource*. In this project, *Software Technologies* were implemented to identify patients with extremely dense breasts who could receive nudges, develop a workflow for pulling EMR details into the text-messaging platform and then delivering the text messages, and to embed clinician-directed messages into mammogram reports. The development of these technological workflows helped expand message reach. Additionally, the project demonstrated *Adaptation* in implementing *Software Technologies.* Different nudge delivery strategies were designed to fit the technical capabilities of different sites, and after seeing low engagement during pilots, the study team added reminders.

This study also demonstrates impact on *Capacity Building* by expanding Penn ISC3 beyond the Cancer Service Line to more than 30 primary care sites. This trial is also co-led by an investigator new to implementation science mentored by the grant’s MPIs. Lastly, the study aims to ensure access for all eligible patients with the goal of doing so equitably across races, thereby reducing barriers to accessing evidence-based interventions consistent with the *Health Care Delivery* and *Health Care Accessibility* TSBM domains.

### Project 4: Evaluating sequential electronic health record based strategies to increase genetic testing for breast and ovarian cancer risk across diverse patient populations in gynecology practices

Breast cancer is the most common cancer among women and ovarian cancer has the highest mortality rate of all cancers for women in the US[51,51]. With advancements in knowledge of the effects of genes on breast and ovarian cancer risk and response to medical treatment, the National Comprehensive Cancer Network recommends testing for specific groups (e.g., those with personal histories of ovarian cancer) [52]. Unfortunately, testing rates are low for women with ovarian cancer or with a family history of ovarian or male breast cancer [53,54]. Further, while mortality rates from breast cancer are higher among Black patients, testing rates are significantly lower, illustrating an important health inequity [55–57].

Clinicians face several barriers to utilizing genomic medicine, including little clarity about patient eligibility, lack of genomic medicine training, lack of time, and concerns about potential patient reactions [58–62]. Likewise, patients report barriers including lack of awareness, inadequate access, concerns about cost, potential misuse of test results, and insurance discrimination, which are higher among racial and ethnic minority groups [63–70]. When perceiving unclear eligibility criteria or facing busy schedules, clinicians may opt to keep things the same to maintain simplicity, falling back on the status quo. Likewise, omission bias, or focusing on the potential harm of action more than that of inaction, may lead patients to avoid genetic testing.

Penn ISC3 launched a study to evaluate three nudges informed by behavioral economics to increase breast and ovarian cancer genetic counseling [71].A pragmatic cohort study design is testing three sequential strategies, two directed at patients (targeting omission bias) and one directed at clinicians (targeting status quo bias), deployed in the EMR for patients in OB-GYN clinics. In the first sequence, a patient portal message is designed as a low-cost method to generate testing interest. Next, similar outreach via text messaging, which has greater reach [72] and may help overcome inequities [73], will be assessed. Lastly, we will send clinician-directed nudges with a default referral for genetic counseling. The primary implementation outcomes are rates of scheduling and completion of genetic counseling appointments. Patient characteristics (e.g., race/ethnicity, nature of genetic risk) will be assessed as moderators of the effectiveness of each sequence to promote genetic testing. Again, the development of this study highlights TSBM domains.

#### TSBM domains

The ability to correctly identify eligible patients was a notable challenge. Thus, supporting the *Methods Development* and *Software Technologies* domains, we developed and validated electronic phenotyping algorithms for identifying patients based on cancer registry data and EMR-based family history fields [74]. As with previous studies, *Rapid Cycle Approaches* were used to quickly learn, innovate, and de-risk our implementation strategies. RCAs involved reviewing prototype messages with clinicians with expertise in breast and ovarian cancer genetic testing, patient partners on the Basser Young Leadership Council, and health system and behavioral design experts. Key informant interviews guided study design and helped refine implementation methods. Two template patient messages were piloted with 200 patients using the patient portal and texting, and the message that patients engaged with most was chosen. Likewise, we created multiple versions of the clinician nudge and ascertained feedback from partners about the content, design, and method of delivery. Under *Software Technologies*, a “pend and send” procedure, newly added to the EMR as part of the health system’s Epic® (Epic Systems Corporation, Verona, WI) upgrade, was implemented to send default orders to clinicians. This offers a simplified workflow for signing orders outside of typical office visits (such as genetic counseling referrals). Procedures for message delivery using this functionality were piloted to ensure operability.

This project addresses two other TSBM domains. First, it is led by two senior investigators new to implementation science. With MPI support, these investigators have developed new expertise in implementation science, thereby supporting *Capacity Building*. In fact, one project lead has subsequently been awarded an NHGRI R01 to expand evaluation of implementation science frameworks for increasing genomic medicine usage across our health system. Lastly, this study is being implemented in two distinct clinics. One serves predominantly white patients, and one serves predominantly Black patients. Given the desire to offer genetic testing to all who could benefit – and doing so equitably – this study aims to assess impact *in Health Care Delivery* and *Health Care Accessibility*.

### Project 5: Behavioral economic strategies to improve PRO adherence

Up to half the time, clinical teams do not recognize the symptoms their patients with cancer are experiencing [75,76]. Monitoring patient-reported outcomes (PROs), involving weekly symptom assessments with care team alerts, has been shown in several clinical trials to reduce care utilization, improve quality of life, and lengthen overall survival [77–79]. Unfortunately, PROs are underused in oncology, especially in real-world settings where adherence is often as low as 50%. Additionally, PRO completion is about 10% lower for nonwhite patients than white patients [80,81].

Multilevel barriers to implementation exist at the patient, clinician, and system levels [82–85]. Current approaches to PRO collection are limited by poor clinician engagement, inability to visualize PRO trends, and insufficient automated effectors actively linking reported symptoms with clinical response. This trial has been designed to overcome these limitations: novel electronic monitoring methods aim to maximize real-time PRO capture and link PRO responses with automated alerts. Using a randomized pragmatic study design, this trial simultaneously evaluates implementation (PRO adherence) and effectiveness (symptom burden) outcomes across three arms: (1) usual care (encounter-based PRO monitoring), (2) usual care plus patient reminders and triage nurse alerts, and 3) remote PRO monitoring plus patient reminders and triage nurse alerts.

#### TSBM domains

Launched in December 2023, this trial is led by the same investigators as project #2 (SICs). They are early-career investigators mentored by the grant’s MPIs, thereby supporting *Capacity Building*. Co-designing strategies and educational presentations with triage nursing have also integrated more people into implementation research and built capacity in *Rapid Cycle Approaches*. The *Adaptation* to include an encounter-based PRO monitoring study arm was guided, in part, by the recognition that remote monitoring may pose challenges to racially and ethnically diverse patients, thereby exacerbating inequities.

Investigators and iLab leadership have engaged in substantial development in *Software Technologies*. First, a survey was implemented for cancer center clinicians. Responses informed the project’s *Methods Development* in optimizing implementation strategies to ensure face validity and maximum effect. Across approximately 100 responses, clinicians were least confident in finding PRO data and pulling them into progress notes, and they indicated that automatic notifications of escalating symptoms and improved PRO visualization would enhance symptom management. In response, new PRO reports were developed in the EMR to visualize trends, an automatic alert system for severe symptoms was created, and a standardized response system was implemented for triage nurses to more easily document patient follow-ups. Additionally, changes were made at the system level, with overall PRO questionnaire updates and a new dot-phrase for clinicians to pull PRO reports. We anticipate that implementation strategies and software technologies developed here will effectively and equitably increase PRO monitoring, increasing *Health Care Delivery* and *Health Care Accessibility*.

## Conclusions

It has become increasingly important for NIH-funded investigators to demonstrate impact beyond traditional metrics, particularly in translational research. Here, we have applied the TSBM to Penn ISC3 to illustrate how researchers can more broadly frame their work’s impact. Some studies have been completed, yielding high-impact publications [24]. However, beyond contributing to scientific literature, our center demonstrates impact across system and societal perspectives. First, we show impact on *Methods Development* (within the Implementation Field) and *Rapid Cycle Approaches.* These concepts borrow from industry, which has learned to “fail fast and learn quickly” through specific innovation methods such as fake back ends (manually testing an implementation strategy prior to automation) or vapor tests (assessing demand using realistic but unavailable services) [17,86]. Paradoxically, RCAs can slow implementation because they can involve iterating through pilot tests and engaging collaborators before scaling an intervention. However, it is crucial to keep endpoints in mind when assessing speed [87], and RCAs can increase the speed at which scientific and translational impact is achieved by increasing buy-in from partners and identifying key pitfalls early. Our application of these methods “de-risked” our interventions, allowing for responsive revision (e.g., modifying alert content and delivery) to increase impact and align with the science of *Adaptation* [88].

Second, we illustrate *Capacity Building* in implementation science research, since all studies are co-led by junior faculty or senior faculty new to implementation science. The Center MPIs consistently mentor these researchers to support their development. This support, in part, helped project leads earn several grants, including an R37 award, an R01, and another P50, and this effort has extended to Northwestern University. Our center has expanded to address practice gaps beyond cancer care and is helping cultivate future generations of interdisciplinary implementation science researchers.

Third, our projects demonstrate impact in *Software Technologies* and the value of embedding implementation strategies in the EMR. New, automated systems increase reach and optimize workflows. The wealth of data within the EMR also facilitates tracking of research outcomes and the identification of potential moderators of effectiveness. Finally, technological developments from Penn ISC3 projects have had system-wide impact, including scaling tobacco use treatment nudges across service lines and upgrading symptom monitoring programs.

Lastly, our goal is to improve the quality and equity of cancer care. Each trial focused on implementation strategies that could eliminate key practice gaps for all patients, including those from racial and ethnic minority groups who may experience the greatest health inequities. Our first two studies have produced implementation strategies that improved rates of tobacco treatment engagement and SICs, and these strategies were equally effective for racial and ethnic minority patients. We anticipate similar impacts on *Healthcare Quality* and *Healthcare Accessibility* from ongoing trials.

### Limitations of TSBM and opportunities for improvement

The TSBM can show how Penn ISC3’s impact extends beyond traditional metrics to include methodological innovations, increased research capacity, and enhanced and equitable cancer care delivery. Nevertheless, although the TSBM can help catalog investigator contributions, it does not offer mechanisms to translate those contributions into recognition or advancement of investigators [1]. Conventional academic promotion processes rely on published manuscripts, invited presentations, and grant support, all of which are easily reported in NIH biosketches and traditional curriculum vitae (CV) formats. However, none of the TSBM domains align with these academic tools. We imagine that few TSBM domains will fit neatly on CVs either, even though CVs are often more inclusive. And of course, documenting impact is only a first step in a process that must include developing conventions and cultures of recognizing those contributions toward academic advancement.

Additionally, as with other implementation science frameworks, the TSBM could be improved by centrally incorporating health equity [89]. As a cross-center product from the ISC3 Health Equity Task Force outlined, strategies for adapting models to explicitly integrate health equity include adding relevant domains, changing definitions of existing constructs, and modifying the organization of domains [90]. The ISC3 network has operationalized these strategies by adding a TSBM indicator focused on expanding research capacity among diverse investigators and developing methods for purposively sampling diverse patients to gather perspectives on barriers and facilitators to trial engagement. Still, more could be done to explicitly incorporate health equity into the TSBM, particularly beyond the Community domain. For instance, Policy (e.g., increasing diversity in expert testimony) and Economic (e.g., ensuring cost-effective interventions, like at-home screening kits, are available for all) indicators could be adapted to focus on health equity.

By being mindful of impacts beyond traditional metrics, and explicitly working towards broader impact from the start, future centers can support the development of their investigators and communities. They also have the potential to create more sustainable impact through improved clinical treatment, public health practice, and policy change. Our hope is that by providing these exemplars demonstrating how our research has broader impacts, other researchers and funding agencies will adopt a comprehensive framework for assessing, recognizing, and rewarding scientific impacts on the lives we work to help.

## Supporting information

Schnoll et al. supplementary material Schnoll et al. supplementary material

## Supplementary material

The supplementary material for this article can be found at https://doi.org/10.1017/cts.2024.554.
