# Supplementary material for: Using the Translational Science Benefits Model to assess the impact of the Penn Implementation Science Center in Cancer Control
Source: J Clin Transl Sci. 2024 Oct 16;8(1):e166. doi: 10.1017/cts.2024.554 (PMC11604509; doi:10.1017/cts.2024.554)
Supplement: Schnoll et al. supplementary material [file S2059866124005545sup001.docx]

**Supplement 1.** Demonstrations of Impact

**Penn ISC3 Project Builds Research Capacity for Triage Nurses**

Nurses and support staff do not always have their voices heard in operational research, even when they are eager to contribute. As part of Project #5, we intentionally integrated a triage nurse into our project team, and we have co-designed triage nurse-directed interventions.

Collaborating with a triage nurse lead has increased buy-in from staff, improving research outcomes and health care quality. Furthermore, the project built capacity and allowed the triage nurse lead to contribute to operational decisions as updates were made to overall PRO monitoring workflows in our cancer center.

**Nudges for Serious Illness Conversations Reveal Hidden Needs**

Dealing with advanced disease can be challenging for patients, and they may not feel comfortable raising concerns to family members or clinicians directly. Our nudges prompting serious illness conversations improved health care quality and accessibility by providing an opening for patients and clinicians to discuss goals of care. One clinician shared an anecdote about their experience:

“[The patient nudge] broke down a barrier that honestly, I hadn’t realized was there, and helped me get [the patient] to a place where she could get a need met that wasn’t getting met before.”

**Tobacco Use Treatment Referrals: Impact at Scale**

Clinician-directed nudges with a default selection **(Figure 1)** increased referral to tobacco use treatment services by three times for patients with cancer. Based on the success of this initiative at equitably impacting health care quality, study investigators are collaborating with health system leadership to expand the nudges’ reach. Work is ongoing to assess clinicians’ perspectives on the nudges and integrate the nudges as a standard of care across the cancer center and in other service lines.
